# Lung cancer biobanking in Australia: challenges and future directions

**DOI:** 10.5694/mja2.70012

**Published:** 2025-07-21

**Authors:** Sarah Yeo, Stephen Q Wong, Farzaneh Atashrazm, Andreas Behren, Anthony T Papenfuss, Natalia Vukelic, Lisa Briggs, Ashleigh R Poh, Daniel Steinfort, Natasha Smallwood, Kate Sutherland, Vivek Naranbhai, Sagun Parakh, Tracy Leong

**Affiliations:** ^1^ University of Melbourne Melbourne VIC; ^2^ Olivia Newton‐John Cancer Research Institute Melbourne VIC; ^3^ Cancer Genomics Translational Research Centre Peter MacCallum Cancer Centre Melbourne VIC; ^4^ Austin Health Melbourne VIC; ^5^ La Trobe University Melbourne VIC; ^6^ Walter and Eliza Hall Institute of Medical Research Melbourne VIC; ^7^ Monash University Melbourne VIC; ^8^ Alfred Hospital Melbourne VIC; ^9^ Royal Melbourne Hospital Melbourne VIC

**Keywords:** Respiratory system, Biological specimen banks, Cancer, Respiratory tract neoplasms, Healthcare disparities

Lung cancer is the leading cause of cancer‐related morbidity and mortality worldwide.^1^ It is the fifth most common cancer diagnosed in Australia, but accounts for almost 20% of all cancer‐related deaths, which is more than breast, prostate and ovarian cancer combined. Global advances in lung cancer management have been driven by early diagnosis, such as lung cancer screening, improved treatment options (targeted therapy and immunotherapy) and improvements in survivorship care. Despite this progress, the five‐year survival rate for lung cancer in Australia is 24%.^2^ Reasons for this poor outlook include late presentation with most lung cancer patients presenting with metastatic disease at the time of diagnosis, as well as tumour‐specific features including a lack of reliable biomarkers, tumour heterogeneity and drug resistance.^3^

Lung cancer biobanks facilitate translational research aimed at improving lung cancer diagnosis, prognosis and treatment in several ways.^4^ These repositories collect and store high quality biological samples, such as tissue and blood, as well as detailed clinical information from patients across various stages of disease. This enables researchers to identify and validate predictive and prognostic biomarkers, discover molecular signatures associated with tumour progression and treatment resistance, and develop novel therapies and strategies to overcome resistance.^5^

This perspective article provides a background on cancer biobanks, outlines the challenges specific to lung cancer biobanks in the Australian context, and discusses future directions.

## Biobanking: an overview

Biobanks are defined by the Organisation for Economic Co‐operation and Development as “a structured resource that can be used for the purpose of genetic research and which include: (a) human biological materials and/or information generated from the analysis of the same; and (b) extensive associated information”.^6^ Cancer‐focused biobanks are invaluable collections of biological specimens accompanied by matched health and demographic data. The International Agency for Research on Cancer states that biobanks underpin three rapidly expanding research fields aimed at developing effective strategies to prevent, diagnose and treat cancer: molecular and genetic epidemiology to assess the genetic and environmental bases of cancer; molecular pathology to develop molecular‐based classifications and further phenotyping of different cancers; and pharmacogenomics/pharmaco–proteomics to understand the correlation between a patient's genotype/phenotype and their response to drug treatment.^7^

## The global landscape in lung cancer biobanking

With the assistance of a medical librarian, a search of Ovid MEDLINE(R) ALL 1946 to 4 April 2025 was conducted using a combination of MeSH headings and keywords containing “lung cancer” and “biobank” (search strategy provided in Supporting Information). There were 488 abstracts identified and screened, resulting in 379 relevant records. Of these, most (316) referred to research conducted using the UK Biobank, underscoring the importance of the UK Biobank in research and clinical contexts. Other cited biobanks included FinnGen (Finland) and China Kadoorie Biobank (China). Very few lung cancer‐specific biobanks were identified, as summarised in Box 1.

Of the lung cancer biobanks described in Box 1, the only biobank currently collecting specimens, as well as being open for researchers’ applications is the Lung Biobank in Heidelberg, which has been operating for over 20 years with 16 000 patient specimens (with most specimens collected from people with lung cancer). Other lung cancer biobanks, such as the Shanghai Chest Hospital Lung Cancer Biobank, the Lung Cancer Biobank Nice and the European Early Lung Cancer Biobank, are no longer collecting specimens, and there is no clear online application pathway for researchers to apply to conduct research on collected specimens. Of these lung cancer biobanks, most lung cancer specimens are from surgical resections, which is concerning as most people with lung cancer have metastatic, unresectable disease, therefore the research produced from resected specimens may not be as relevant.

BOX 1 **Summary of collection and processing methods for lung cancer tissue in lung cancer biobanks worldwide**

**Table:** 

Biobank	Country	Number of patients	Method of lung cancer tissue collection	Lung tissue processing method	Other samples collected	Ongoing tissue collection and open for research applications
Lung Biobank Heidelberg^8^	Germany	~10 000	Surgical resection, CT biopsy, bronchoscopy	FFPE, cryopreserved	Matched normal tissue, blood, breath condensate, urine	Yes
Shanghai Chest Hospital Lung Cancer Biobank^9^	China	2000	Surgical resection	FFPE	Blood, pleural effusions	No
Southern Chinese Lung Cancer Biobank^10^	China	1054	Surgical resection	FFPE, cryopreserved	Matched normal tissue, blood	No
Lung Cancer Biobank Nice^11^	France	3798	Surgical resection	FFPE, cryopreserved	Matched normal tissue, blood	No
LUNGBANK^12^	Turkey	237	Surgical resection	Homogenised into a powder, immersed in tissue culture then minced and frozen	Nodal tissue, reactive stroma, blood, urine, stool, sputum, BALF	No
University of Torino Lung Cancer Biobank^13^	Italy	135	Surgical resection	FFPE, cryopreserved	Blood, saliva	No
European Early Lung Cancer Biobank^14^	Europe	1176	Surgical resection	FFPE, cryopreserved	Blood, sputum, BALF, matched normal lung tissue	No

BALF = bronchoalveolar lavage fluid; CT = computed tomography; FFPE = formalin‐fixed paraffin‐embedded.

Australia is home to several well established pan‐cancer biobanks as well as tumour‐specific biobanks, but lung cancer biobanking in Australia is in its infancy. An example of an Australian cancer‐agnostic biobank is the Victorian Cancer Biobank — which holds over 460 000 biospecimens — and provides access to samples collected in Victoria for approved research projects. The Victorian Cancer Biobank includes lung cancer specimens and as of April 2025, its catalogue listed lung cancer resection specimens from more than 50 donors and four metastatic lymph node specimens from two donors with advanced lung cancer.^15^ There are also Australian cancer‐specific biobanks, such as the Melanoma Institute Australia Biospecimen Bank, the Australian Breast Cancer Tissue Bank, and Brain Cancer Biobanking Australia. In addition, the new TRACKER (Tissue Repository of Airway Cancers for Knowledge Expansion of Resistance) lung cancer biobank is a consumer‐partnered initiative focused on lung cancer.^16^ TRACKER stores longitudinal biospecimens (including tumour tissue, peripheral blood and bronchoalveolar lavage fluid [BALF]) at diagnosis, during therapy and at disease progression.

## Tissue is the issue

Traditionally, lung cancer tissue for biobanking has been acquired through surgical resection of early‐stage tumours, as this method provides large samples suitable for analysis. However, surgical biopsies are not representative of tumours in most individuals diagnosed with lung cancer, as most patients present with metastatic disease, which is inoperable.^17^ Tissue acquisition from metastatic sites is often complex due to anatomical inaccessibility or patient‐related factors and, as such, staging metastatic lung cancer can be challenging.^18^ An example of a challenging metastatic site is the brain, which is difficult to obtain samples from without resection and usually carries high peri‐operative risk. Similarly, liver and adrenal biopsies pose risks such as bleeding or bile leakage due to proximity to vascular structures, whereas bone biopsies are often painful and may not yield sufficient tissue for diagnosis.

Often the most practical methods for obtaining tissue for both diagnostic and biobanking purposes are minimally invasive bronchoscopic techniques such as linear endobronchial ultrasound‐guided transbronchial needle aspiration (EBUS‐TBNA) for accessible nodal disease, and computed tomography‐guided lung biopsies for direct sampling of lung lesions. Cytological specimens obtained from linear EBUS‐TBNA procedures have been shown to provide adequate DNA yields for molecular analyses, including targeted and whole exome sequencing.^19^ It is well established that the tumour microenvironment varies depending on anatomical site of disease.^20^ Therefore, to facilitate the study of heterogenous responses to treatment, a biobank should include a range of anatomical samples.

Longitudinal sampling is crucial in lung cancer research due to the dynamic nature of biomarkers (eg, programmed death ligand‐1; PD‐L1), which vary both spatially (depending on the biopsy site) and temporally (changing throughout treatment). As immunotherapy efficacy is closely linked to PD‐L1 expression levels, understanding resistance mechanisms necessitates repeated tissue sampling across different treatment phases. Minimally invasive procedures, such as EBUS‐TBNA, allow for longitudinal sampling and can provide invaluable insights into tumour evolution, resistance mechanisms and patient‐specific responses to therapies.^8^

A key challenge with longitudinal sampling is the ethical and clinical concern of subjecting patients to repeated procedures purely for research purposes, which may increase patient risk and compromise ethical standards. When tissue is required for multiple purposes, including: (i) diagnosis; (ii) molecular analysis to guide treatment decisions; (iii) biobanking for future research; and (iv) eligibility for clinical trial enrolment, a single sample may not be sufficient to meet all these demands. Engaging with organisations that can facilitate access to archived tissue samples, or through participation in national networks, such as Lung Foundation Australia, the Thoracic Oncology Group Australasia, the Thoracic Society of Australia and New Zealand, and the Australian Registry and Biobank of Thoracic Cancers, allows for research collaboration, which may improve analysis methods so more can be done with less tissue.

Blood‐based liquid biopsies offer a less invasive alternative for tracking circulating tumour DNA throughout the course of treatment, enabling monitoring of genomic alterations associated with therapy resistance.^21^ Measuring circulating tumour DNA to assess tumour response is particularly valuable in lung cancer, where repeat tissue biopsies are often impractical. This approach allows for more refined prognostic assessments, tailored surveillance intervals and informed adjustments to therapy. Biobanks that collect a variety of lung cancer biospecimens longitudinally will be critical for translational research, helping to drive the development of personalised treatment strategies based on dynamic molecular profiling.

## Research applications for biobanked lung cancer specimens

Translational research using biobanked lung cancer specimens encompasses a range of analytical approaches. These include genomic analysis (eg, identifying mutations such as in epidermal growth factor receptor [*EGFR*]), transcriptomic profiling (eg, assessing PD‐L1 expression, which can correlate with immunotherapy response), and microbiome studies (eg, understanding how gut bacteria influence treatment outcomes). When these layers of data are combined, the approach is referred to as multi‐omics, enabling comprehensive molecular profiling that supports the development of personalised treatment strategies tailored to the evolving nature of an individual's disease.

Integrating multi‐omic data provides deeper insights into resistance mechanisms. This strategy can identify predictive biomarkers for systemic therapies as well as potential applications in surgical and radiotherapy interventions in future studies.

## Unique challenges of lung cancer biobanking in Australia and future directions

Internationally, efforts in achieving proportional ethnic diversity in biobanks have been driven by legislation, such as: (i) setting recruitment guidelines from the outset to ensure diversity and developing culturally competent guidelines for researchers; (ii) establishing ethnic diversity recruitment subgroups to broaden participation; and (iii) creating culturally informed research frameworks. In contrast, Australia lacks a legislative framework to guide the inclusion of Indigenous peoples and other ethnically diverse populations in biobanks or biomedical research.^22^ Additional barriers for these priority populations, particularly Indigenous communities, include concerns surrounding the collection, storage and use of biological specimens, as well as uncertainties related to data sharing and ownership rights. Research involving Indigenous Australians must adapt study protocols to respect cultural values, address community priorities, ensure meaningful community consultation and include Indigenous researchers.^23^

To enhance the relevance and impact of biobanks in health and medical research, it is vital to engage with patients, caregivers and consumer advocates. The stakeholder insights and feedback help shape study design, while participant information and consent processes ensure that research is patient‐centred and reflective of real‐world needs. Their involvement also strengthens advocacy efforts and recruitment strategies, fostering broader community trust and participation in research initiatives. By prioritising consumer engagement, we can build a research environment that authentically reflects the experiences and priorities of those it seeks to benefit.

Despite increasing efforts to support inclusive participation, significant gaps remain. There is a lack of diversity among consumers involved in research, echoing similar challenges seen in the composition of biobanked specimens. Priority populations — including populations with lower levels of health literacy, education and socio‐economic status — continue to be under‐represented.^24^ This disparity persists despite the existence of national frameworks such as the National Framework for Consumer Involvement in Cancer Control.^25^

The translational potential of biobanks depends on access to researchers who can effectively use these biospecimens for high quality analyses and publication in peer‐reviewed journals, with the goal to improve outcomes of people with lung cancer (Box 2).

Box 2 Key components and workflow of lung cancer biobanking*

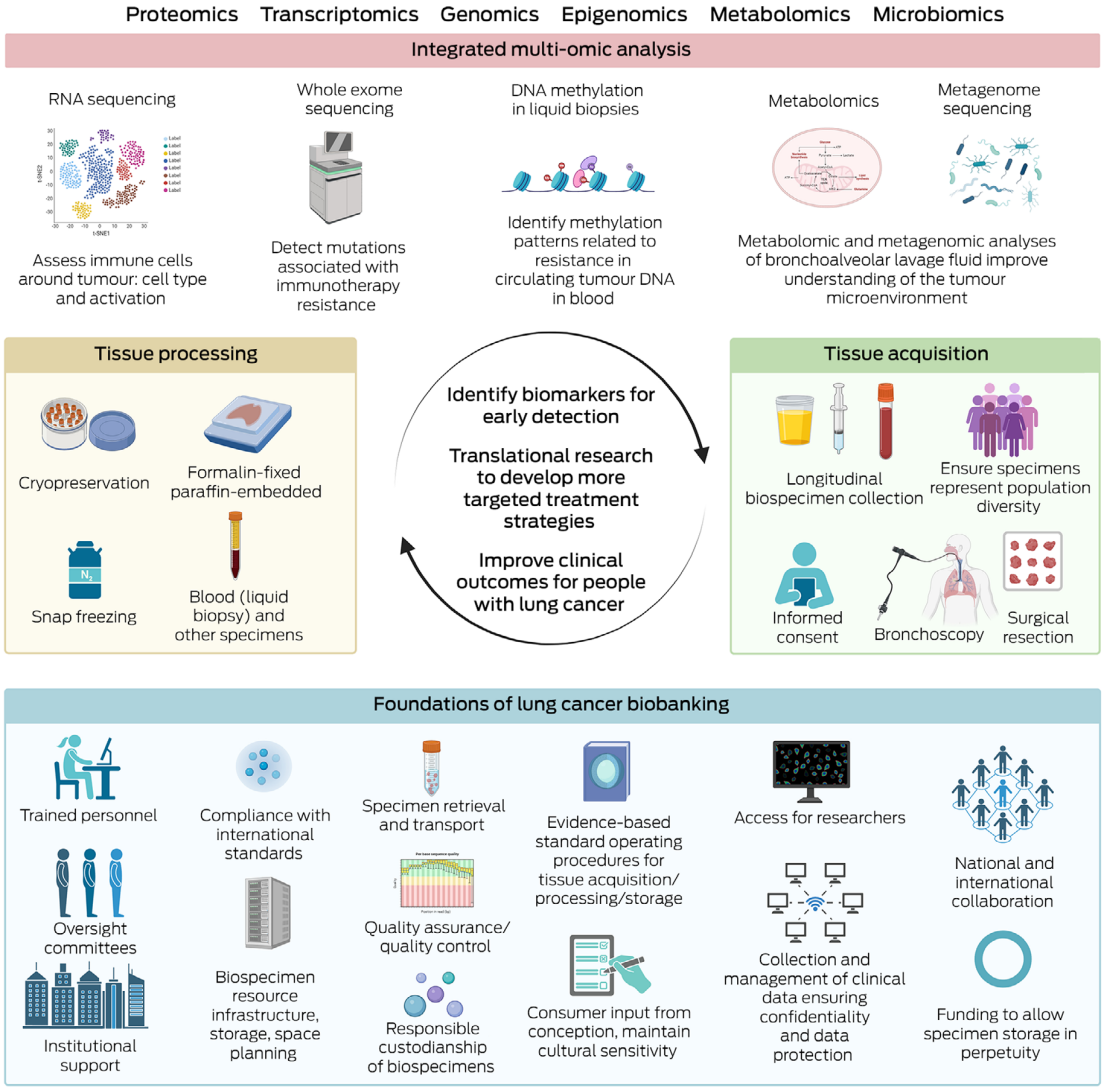

* This figure details foundational components, tissue acquisition/processing techniques, and analyses that can be used for lung cancer biobanking, aiming to identify biomarkers for early detection/treatment resistance and develop targeted treatments to improve clinical outcomes for people with lung cancer. Created with BioRender.com.

## Conclusion

Despite considerable advances in lung cancer management, significant challenges remain in improving patient outcomes. High quality, long term, consumer‐partnered lung cancer biobanks are critical to advancing the necessary translational research.

The development of large, clinically annotated, multi‐omic datasets from lung cancer patients will provide essential insights into predictive and prognostic biomarkers and identify novel therapeutic targets to overcome resistance and improve treatment responses. Enhancing patient selection for clinical trials and identifying new agents to target resistance mechanisms may enable more personalised therapies. This will facilitate the design of synergistic combination strategies for treatment‐refractory patients.

Reducing treatment resistance will ultimately lead to better clinical outcomes, improved survival and enhanced quality of life for patients with lung cancer. Lung cancer biobanking is a crucial step in building the infrastructure needed to enable access to biospecimens and associated multi‐omic and clinical data for researchers both nationally and internationally. This will increase research efficiency, reduce costs and, most importantly, foster greater collaboration, all with the goal of improving the lives and outcomes of people affected by cancer.

## Open access

Open access publishing facilitated by The University of Melbourne, as part of the Wiley ‐ The University of Melbourne agreement via the Council of Australian University Librarians.

## Competing interests

No relevant disclosures.

## Provenance

Not commissioned; externally peer reviewed.

## Author contribution statement

Yeo S: Investigation, methodology, visualization, writing ‐ original draft, writing ‐ review and editing. Wong SQ: Writing ‐ review and editing. Atashrazm F: Resources, writing ‐ review and editing. Behren A: Conceptualization, writing ‐ review and editing. Papenfuss AT: Writing ‐ review and editing. Vukelic N: Project administration, resources, writing ‐ original draft, writing ‐ review and editing. Briggs L: Conceptualization, writing ‐ review and editing. Poh AR: Writing ‐ review and editing. Steinfort D: Supervision, writing ‐ review and editing. Smallwood N: Supervision, writing ‐ review and editing. Sutherland K: Supervision, writing ‐ review and editing. Naranbhai V: Supervision, writing ‐ review and editing. Parakh S: Conceptualization, supervision, writing ‐ original draft, writing ‐ review and editing. Leong T: Conceptualization, supervision, writing ‐ original draft, writing ‐ review and editing.

## Supporting information


**Data S1** Supplementary Table
