# Supplementary material for: Lung cancer biobanking in Australia: challenges and future directions
Source: Med J Aust. 2025 Jul 21;223(4):180–4. doi: 10.5694/mja2.70012 (PMC12358101; doi:10.5694/mja2.70012)
Supplement: Supplementary file 1 — Data S1 Supplementary Table [file MJA2-223-180-s001.pdf]

Supporting Information

Supplementary material

This appendix was part of the submitted manuscript and has been peer reviewed. It is posted as supplied by the authors.

Appendix to: Yeo S, Wong SQ, Atashrazm F, et al. Lung cancer biobanking in Australia: challenges and future directions. *Med J Aust* 2025; doi: 10.5694/mja2.70012.

Summary of Search Strategy

Ovid MEDLINE(R) ALL <1946 to April 04, 2025>

#	Query	Results from 7 Apr 2025
**1**	lung neoplasms/ or "adenocarcinoma of lung"/ or adenocarcinoma, bronchiolo-alveolar/ or bronchial neoplasms/ or carcinoma, bronchogenic/ or carcinoma, non-small-cell lung/	292 476
**2**	((lung or pulmonary) adj3 (neoplas* or cancer* or carcinoma* or malignan* or metastat* or tumo?r*)).ti,ab,kf.	307 343
**3**	1 or 2	405 101
**4**	biological specimen banks/ or tissue banks/	11 422
**5**	UK Biobank/	775
**6**	biological specimen bank*.ti,ab,kf.	83
**7**	biobank*.ti,ab,kf.	21 214
**8**	biorepositor*.ti,ab,kf.	1371
**9**	bio-bank*.ti,ab,kf.	241
**10**	bio-repositor*.ti,ab,kf.	58
**11**	tissue bank*.ti,ab,kf.	2537
**12**	4 or 5 or 6 or 7 or 8 or 9 or 10 or 11	29 578
**13**	3 and 12	488
